# Optimal approach for diagnosing peripheral lung nodules by combining electromagnetic navigation bronchoscopy and radial probe endobronchial ultrasound

**DOI:** 10.1111/1759-7714.15376

**Published:** 2024-06-17

**Authors:** Bora Lee, Hee Sang Hwang, Se Jin Jang, Sang Young Oh, Mi Young Kim, Chang‐Min Choi, Wonjun Ji

**Affiliations:** ^1^ Division of Pulmonology and Critical Care Medicine, Department of Internal Medicine, Asan Medical Center University of Ulsan College of Medicine Seoul Korea; ^2^ Department of Pathology, Asan Medical Center University of Ulsan College of Medicine Seoul South Korea; ^3^ Department of Radiology, Asan Medical Center University of Ulsan College of Medicine Seoul South Korea; ^4^ Department of Oncology, Asan Medical Center University of Ulsan College of Medicine Seoul South Korea

**Keywords:** combination, electromagnetic navigation bronchoscopy, endobronchial ultrasound, peripheral lung lesion, radial probe

## Abstract

**Introduction:**

Electromagnetic navigation bronchoscopy (ENB) and radial probe endobronchial ultrasound (RP‐EBUS) are essential bronchoscopic procedures for diagnosing peripheral lung lesions. Despite their individual advantages, the optimal circumstances for their combination remain uncertain.

**Methods:**

This single‐center retrospective study enrolled 473 patients with 529 pulmonary nodules who underwent ENB and/or RP‐EBUS biopsies between December 2021 and December 2022. Diagnostic yield was calculated using strict, intermediate, and liberal definitions. In the strict definition, only malignant and specific benign lesions were deemed diagnostic at the time of the index procedure. The intermediate and liberal definitions included additional results from the follow‐up period.

**Results:**

The diagnostic yield of the strict definition was not statistically different among the three groups (ENB/Combination/RP‐EBUS 63.8%/64.2%/62.6%, *p* = 0.944). However, the diagnostic yield was superior in the ENB + RP‐EBUS group for nodules with a bronchus type II or III and a solid part <20 mm (odds ratio 1.96, 95% confidence interval 1.09–3.53, *p* = 0.02). In terms of complications, bleeding was significantly higher in the ENB + RP‐EBUS group (ENB/Combination/RP‐EBUS 3.7% /6.2/0.6%, *p* = 0.002), but no major adverse event was observed.

**Conclusion:**

The combination of ENB and RP‐EBUS enhanced the diagnostic yield for nodules with bronchus type II or III and solid part <20 mm, despite a slightly elevated risk of bleeding. Careful patient selection based on nodule characteristics is important to benefit from this combined approach.

## Graphical abstract

Combining electromagnetic navigation bronchoscopy withradial probe endobronchial ultrasound enhanced the diagnostic yield in casesinvolving nodules where the solid portion is smaller than 20 mm and thebronchus does not terminate directly into the nodule.

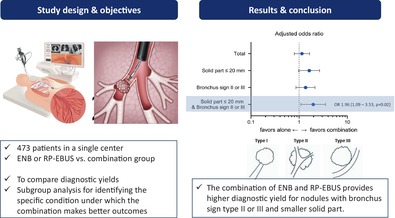

## INTRODUCTION

The widespread use of low‐dose chest computed tomography (CT) for lung cancer screening has led to the early detection of many lung nodules, especially in the peripheral lung fields.^1^, ^2^, ^3^, ^4^ A widely used biopsy procedure for peripheral lung nodules is percutaneous transthoracic needle biopsy (PCNB), with a sensitivity, specificity, and adequacy greater than 90%.^5^ However, its diagnostic accuracy is reportedly reduced to 78.8% for small pulmonary nodules measuring ≤10 mm.^6^ In addition, PCNB has the limitation of having relatively high complication rates, reported up to 38.8% in one meta‐analysis, and is more risky in emphysematous lung, which is not rare in patients with lung cancer.^7^, ^8^ Surgical resection is another option because of its extremely high diagnostic accuracy and can also be used to treat early lung cancer. However, it is the most invasive diagnostic tool, so the risk and severity of complications are high.^9^ Clinicians therefore often hesitate to recommend surgical resection, especially for small lung nodules, which are mostly benign. Recently, several advanced bronchoscopic procedures with relatively low complication rates have been introduced for the diagnosis of lung nodules, such as electromagnetic navigational bronchoscopy (ENB), radial probe endobronchial ultrasound (RP‐EBUS), and robotic‐assisted bronchoscopy.^9^, ^10^, ^11^

ENB is an image‐guided bronchoscopic technique based on electromagnetic navigational assistance. Using information obtained from CT scans and electromagnetic sensors, ENB reconstructs the tracheobronchial tree and visualizes the exact airway to the target lesion.^9^ Another technique is RP‐EBUS, which uses a special catheter to provide a 360° ultrasound view through the working channel of the bronchoscope. This allows an operator to confirm whether the target has been reached by providing ultrasound images.^12^ Combining ENB and RP‐EBUS seems beneficial because it enables the performer to determine the correct bronchus, approach the lesion using ENB, and then confirm the lesion using RP‐EBUS.^13^ Most previous studies have shown consistent results, suggesting that combining the two methods would be advantageous.^14^, ^15^, ^16^ However, increased procedure time, cost, and complications should be considered. Therefore, it is crucial to identify the characteristics of nodules that would benefit from the combination of ENB and RP‐EBUS for accurate diagnosis. In this study, the diagnostic yield and safety profiles of ENB, ENB + RP‐EBUS, and RP‐EBUS were evaluated. The characteristics of nodules that would benefit from the combination of ENB and RP‐EBUS were also identified.

## METHODS

### Study population

This retrospective, single‐center study enrolled 473 patients with peripheral pulmonary nodules who underwent ENB or RP‐EBUS at the Asan Medical Center (Seoul, Republic of Korea) between December 2021 and December 2022.

Patients with inaccessible pulmonary nodules or a high risk of complications from PCNB were included. Patients who did not obtain diagnostic results from PCNB were also included. The exclusion criteria were as follows: (1) bronchoscopy was not performed for diagnostic purposes, (2) bronchoscopy failed to approach pulmonary nodules, and (3) absence of a medical record.

### Clinical assessment

Clinical data, including age, sex, height, body weight, smoking history, and results of pulmonary function tests were reviewed from medical records. Chest CT scans performed within 28 days before bronchoscopy were reviewed to assess the location and characteristics of the pulmonary nodules. The location of the nodule was determined by its proximity to the hilum and the costal surface within the transverse plane and was categorized into three sections: I (inner 1/3), II (middle 1/3), and III (outer 1/3). All nodules were assessed for bronchus signs, and if present, they were categorized into three types.^17^ The type I bronchus ends directly into the lesion, the type II bronchus traverses within the lesion, and the type III bronchus is extrinsically compressed and bent by the lesion. Two reviewers independently determined the type of bronchus, and disagreements were resolved through discussion. All types of procedure‐related complications were defined on the basis of their severity and clinical interventions required. Mild bleeding was defined as spontaneous stoppage during bronchoscopy. Moderate bleeding was defined as the need for cold saline, topical vasoconstrictive drugs, or intravenous tranexamic acid. Severe bleeding was defined as the need for transfusion for invasive interventions, such as arterial embolization, balloon tamponade, or mechanical ventilation. After bronchoscopy, all patients underwent chest x‐rays to confirm the presence of pneumothorax. The need for a chest tube was also identified.

### Bronchoscopic procedures

Before the procedure, the performing bronchoscopist decided between ENB and RP‐EBUS as the main technique. If ENB was selected and the lesion was navigated and approached, the additional use of a radial probe to confirm the target lesion location was at the discretion of the performing bronchoscopist. If RP‐EBUS was chosen initially, only RP‐EBUS was performed. All procedures were performed under moderate conscious sedation using midazolam and fentanyl. For ENB, the SPiN Thoracic Navigation System (SYS‐4230 K; Veran Medical) was used. For RP‐EBUS, a 1.4‐mm UM‐S20‐17S probe (Olympus Corporation) was used as the ultrasonographic probe. For both techniques, a flexible bronchoscope with an outer diameter of 4 mm (P260F) or 6 mm (1 T260) (Olympus Corporation) was used. The performer mostly used forceps to perform biopsies. However, needle aspiration was performed for lesions with no distinct bronchus sign but close to the bronchus. Rapid on‐site examination and fluoroscopy were not performed in this study.

### Defining the results of the procedures

At the time of index bronchoscopy, the pathologic results of the biopsies were initially categorized into four groups: (1) malignancy, (2) nonmalignant histopathology leading to a specific diagnosis of benign disease, (3) nonmalignant but nonspecific benign findings (e.g., chronic inflammation), and (4) nondiagnostic findings (e.g., normal bronchus, normal lung parenchyma, or exudative/necrotic tissue only).^18^ Following a minimum follow‐up of 6 months, the biopsy results were reclassified into five categories: (1) malignancy, (2) cases treated as malignancy without a confirmed diagnosis because of high suspicion of malignancy, (3) specific diagnosis of benign disease, (4) nonspecific benign disease, (5) nondiagnostic findings, and (6) indeterminate (patients without changes in imaging during the follow‐up period or loss to follow‐up).

The diagnostic yield was calculated using three definitions: strict, intermediate, and liberal. The strict definition included all malignant and specific benign lesions as diagnostic at the time of bronchoscopy. The results available only at the time of the procedure were reflected in the strict definition. In addition to the strict definition, the intermediate definition also classified nonspecific benign lesions (excluding nondiagnostic results such as normal lung parenchyma) as diagnostic if malignancy was not confirmed after at least 6 months of follow‐up. Indeterminate cases were regarded as nondiagnostic. The liberal definition considered all nonspecific benign lesions at the index procedure with no malignancy at follow‐up as diagnostic, excluding indeterminate cases. Patients treated for malignancy without a confirmed diagnosis were excluded from the calculation of diagnostic yields. Diagnostic yields calculated using the strict definition were used for logistic regression models and subgroup analysis.

According to the guidance of reporting diagnostic testing suggested by Ost et al. and the list of diagnoses from the AQuIRE registry, the bronchoscopy findings were constructed into a contingency table.^16^, ^19^

### Statistical analysis

Categorical variables are expressed as absolute counts and relative frequencies. Continuous variables are presented as means ± standard deviations or medians (ranges). The diagnostic yield for each bronchoscopic technique was calculated as the number of diagnostic procedures divided by the sum of nondiagnostic and diagnostic procedures. The Kolmogorov–Smirnov test was used to test data normality. A two‐tailed chi‐squared test and Fisher's exact test with a 5% level of significance were used to compare the diagnostic yields. Multivariable logistic regression models were used to identify the effects of the factors on diagnostic yields. The IBM Statistical Package for the Social Sciences (version 24.0; SPSS Inc.) and the R Statistical Package (version 4.3.0; R Foundation for Statistical Computing) were used for data analysis.

## RESULTS

### Study population

A total of 518 patients underwent ENB, RP‐EBUS, or ENB + RP‐EBUS at the Asan Medical Center (Seoul, South Korea) between December 2021 and December 2022. Among them, 45 patients were excluded as per the exclusion criteria, and 473 patients with 529 pulmonary nodules were included in the final analysis. The patients' mean age was 67.2 ± 11.0 years, and 58.8% (278/473) were male. There was no difference in the patients' baseline characteristics among the ENB alone, RP‐EBUS alone, and combination groups (Table 1).

**TABLE 1:** Baseline characteristics of patients

	ENB	Combination	RP‐EBUS	*p* value
Number of patients	107	194	172	
Age, years	66.0 ± 11.8	67.4 ± 10.1	67.6 ± 11.5	0.469
Male (%)	58 (54.2)	112 (57.7)	108 (62.8)	0.341
Height, cm	161.4 ± 8.8	161.7 ± 10.7	162.5 ± 8.1	0.622
Smoking				0.553
Never	57 (53.3)	108 (55.7)	93 (54.1)	
Ex‐smoker	29 (27.1)	50 (25.8)	55 (32.0)	
Current smoker	21 (20.3)	36 (18.6)	24 (14.0)	
Pulmonary function				
FEV_1_, % predicted	86.0 ± 15.3	83.9 ± 18.4	85.4 ± 18.0	0.919
D_LCO_, % predicted	79.3 ± 18.4	76.9 ± 18.6	73.8 ± 21.2	0.118

Abbreviations: D_LCO_, diffusing capacity for carbon monoxide; ENB, electromagnetic navigation bronchoscopy; FEV_1_, forced expiratory volume in 1 s; RP‐EBUS, radial probe endobronchial ultrasound.

### Characteristics of the nodules

Table 2 shows the distribution and characteristics of the nodules. Approximately 64.5% of the nodules (341/529) were solid, and 97.9% had a visible bronchus sign on CT, of which 35.5% (184/518) were type I. Approximately 47.8% of the nodules (253/529) were located in both upper lobes, whereas 43.7% (231/529) were located in both lower lobes. The location, type of bronchus sign, and solidity of the nodules were not different among the three groups. However, the mean total size and solid part size of the nodules were smaller in the combination group than in the alone groups (mean size 25.9 mm in combination vs. 27.9 mm in ENB vs. 31.2 mm in RP‐EBUS, *p* < 0.001).

**TABLE 2:** Distribution and characteristics of pulmonary nodules

	ENB	Combination	RP‐EBUS	*p* value
Total number of nodules	130	212	187	
Location (lobe)				0.093
Right upper lobe	35 (26.9)	53 (25.0)	50 (26.7)	
Right middle lobe	9 (6.9)	13 (6.1)	23 (12.3)	
Right lower lobe	28 (21.5)	50 (23.6)	47 (25.1)	
Left upper lobe	36 (27.7)	42 (19.8)	37 (19.8)	
Left lower lobe	22 (16.9)	54 (25.5)	30 (16.0)	
Location (central vs. peripheral)				0.571
I	35 (26.9)	50 (23.6)	53 (28.3)	
II	40 (30.8)	59 (27.8)	45 (24.1)	
III	55 (42.3)	103 (48.6)	89 (47.6)	
Distance from visceral pleural, mm	18.3 ± 15.1	16.0 ± 13.9	15.1 ± 13.3	0.119
Size, mm	27.9 ± 14.5	25.9 ± 10.9	31.2 ± 13.1	<0.001
Size of solid portion, mm	21.7 ± 13.9	20.2 ± 11.3	26.7 ± 14.1	<0.001
Type				0.303
Solid	80 (61.5)	130 (61.3)	131 (70.1)	
Part‐solid	44 (33.8)	76 (35.8)	51 (27.3)	
Ground‐glass opacity	6 (4.6)	6 (2.8)	5 (2.7)	
Bronchus sign	122	204	185	0.112
Type I	49 (38.6%)	64 (30.9%)	71 (38.2%)	
Type II	36 (28.3%)	77 (37.2%)	71 (38.2%)	
Type III	41 (32.3%)	66 (31.9%)	44 (23.7%)	
Metabolic activity in PET, mean SUV_max_	5.0 ± 4.0 (*n* = 103)	4.4 ± 4.1 (*n* = 191)	4.9 ± 4.1 (*n* = 158)	0.429

Abbreviations: ENB, electromagnetic navigation bronchoscopy; PET, positron emission tomography; RP‐EBUS, radial probe endobronchial ultrasound; SUV_max_, the maximum standardized uptake value.

### Diagnostic yields

**FIGURE 1:**
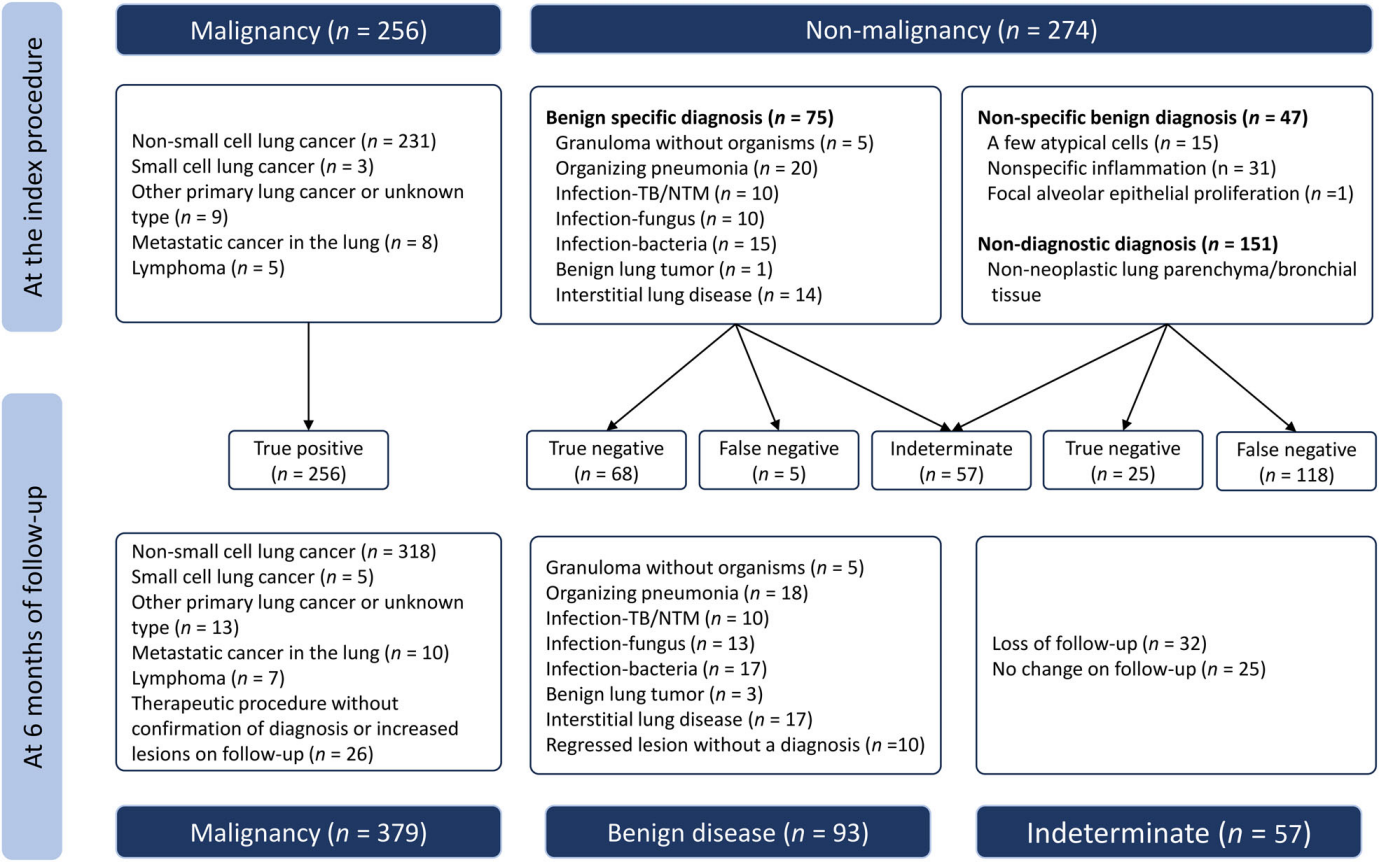
Diagnostic outcomes at the time of bronchoscopic procedures and follow‐up. NTM, nontuberculous mycobacteria; TB, tuberculosis.

The diagnostic results of all cases are presented in Figure 1, and a contingency table of diagnoses was constructed (Supporting Information Table S1). The index procedure diagnosed malignancy in 256 of 529 patients (48.4%). After 6 months of follow‐up, among the 273 initially nonmalignant cases 93 (34.1%) were considered true negatives and 123 (45.1%) were considered false negatives. Table 3 compares the diagnostic yields calculated using the three methods. In all definitions, the diagnostic yields were indifferent among the three groups (63.8% in the ENB group vs. 64.2% in the combination group vs. 62.6% in the RP‐EBUS group, *p* = 0.944). According to the liberal definition, the diagnostic yields of the ENB, combination, and RP‐EBUS groups increased to 79.7%, 75.4%, and 77.1%, respectively.

**TABLE 3:** Diagnostic yields and total procedure time for each group

	ENB	Combination	RP‐EBUS	*p* value
Strict	83/130 (63.8)	136/212 (64.2)	117/187 (62.6)	0.944
Intermediate	86/127 (67.7)	128/199 (64.3)	120/177 (67.8)	0.727
Liberal	94/118 (79.7)	138/183 (75.4)	131/170 (77.1)	0.693
Total procedure time (min)	15.8 ± 3.6	16.0 ± 4.1		0.539

Abbreviations: ENB, electromagnetic navigation bronchoscopy; RP‐EBUS, radial probe endobronchial ultrasound.

### Logistic regression analyses of the diagnostic yield and subgroup analyses

**FIGURE 2:**
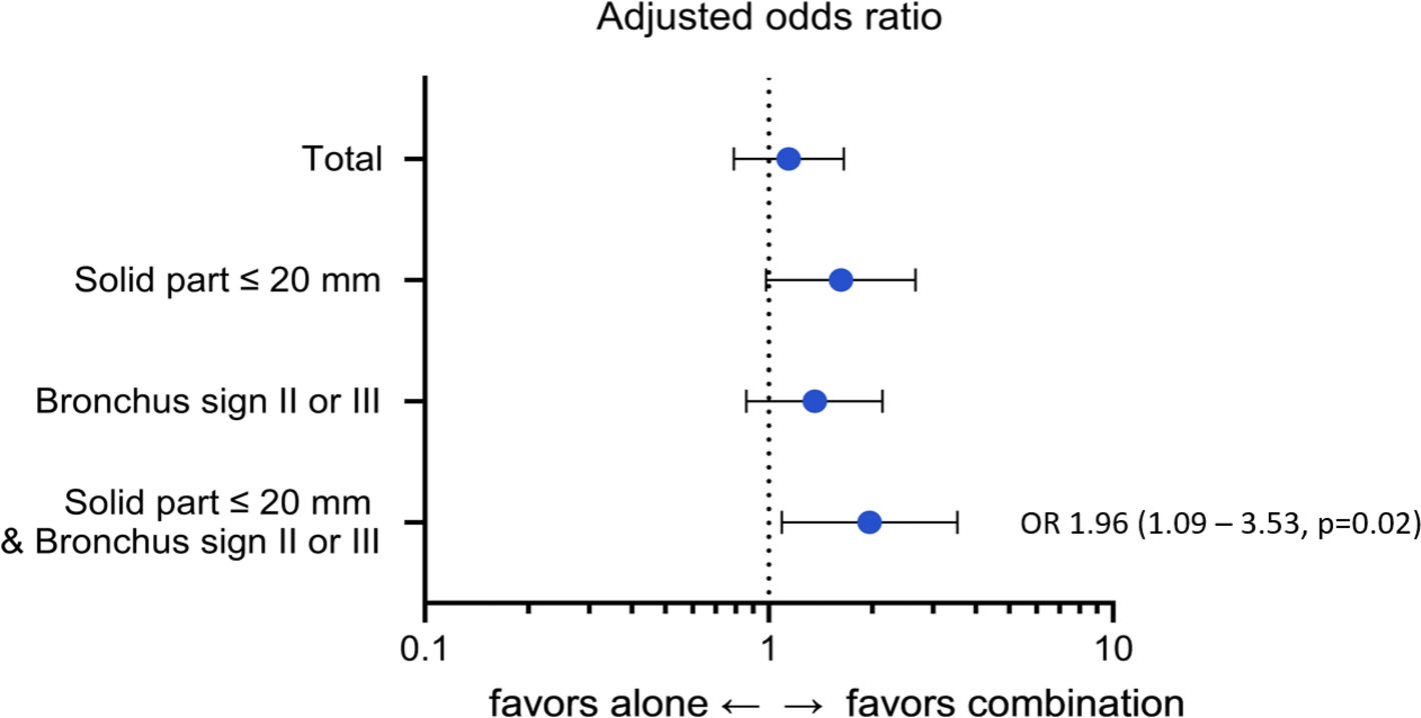
Forest plot from multivariable subgroup analyses. †Size of the solid part, type of bronchus sign, solidity of the nodule and class of the peripheral location were included in the multivariable analysis. OR, odds ratio.

**FIGURE 3:**
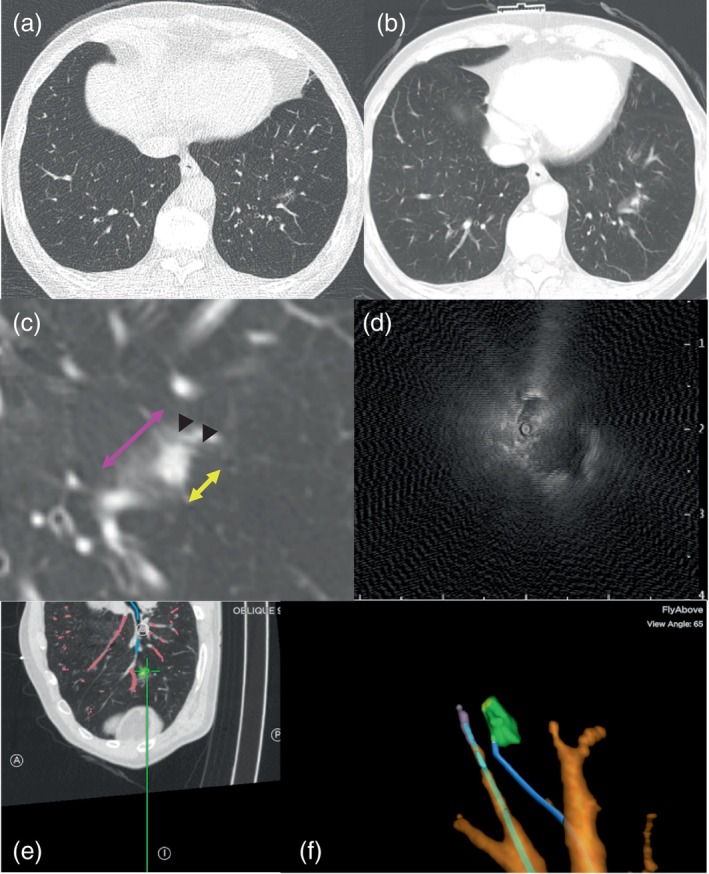
Example of a part‐solid nodule with a solid component smaller than 20 mm and bronchus type III. Compared with the CT findings from 1 year prior (a), the nodule in the left lower lobe had increased in size (b) and malignancy was highly suspected. However, the lung function of the patient was poor, making surgical resection infeasible, therefore a combination of ENB and RP‐EBUS was performed for a biopsy of the lesion. (c) The bronchus transversing around the nodule (arrowheads), a 9.6‐mm solid component (yellow arrow), and a 21.4‐mm whole lesion (pink arrow). (d) The eccentric nodule on RP‐EBUS. (e) The ENB system showing the nodule as the scope approaches. (f) The scope reaching the nodule. The patient was diagnosed with adenocarcinoma and received stereotactic body radiotherapy.

Multivariable analysis showed that the size of the solid part larger than 20 mm was associated with a higher diagnostic yield (odds ratio 1.62, 95% confidence interval [CI] 1.08–2.43, *p* = 0.02) (Supporting Information Table S2). The bronchus sign type II or III tended to be associated with a lower diagnostic yield, although statistically insignificant, and no other variables were associated with diagnostic yield (Supporting Information Table S2). Subgroup analyses were performed based on the factors identified to be associated with lower diagnostic yield in the multivariable analysis. The combination of ENB and RP‐EBUS was associated with a higher diagnostic yield than the alone groups, with nodules less than 20 mm and a bronchus sign of type II or III (odds ratio 1.96, 95% CI 1.09–3.53, *p* = 0.02) (shown in Figure 2). Figure 3 shows typical CT and RP‐EBUS images of a nodule with a solid part less than 20 mm and bronchus sign type II or III. When we selected only solid nodules, the combination did not appear to be beneficial for enhancing diagnostic yields (Supporting Information Table S3A). However, when nonsolid nodules were selected, the combination improved the diagnostic yield by an odds ratio of 2.56 (95% CI 1.34–4.89, *p* = 0.004), and solid components exceeding 20 mm were also associated with increased diagnostic yields (Supporting Information Table S3B).

### Safety profiles

Table 4 presents the procedure‐related complications. Pneumothorax was the most common complication in the groups, and the rate of pneumothorax did not differ among the three groups. However, the bleeding rate was significantly higher in the combination group (6.2% in combination vs. 3.7% in ENB vs. 1% in RP‐EBUS, *p* = 0.002), but no severe bleeding was observed in any group.

**TABLE 4:** Safety profiles of each group

	ENB (%)	Combination (%)	RP‐EBUS (%)	*p* value
Pneumothorax	5 (4.7)	14 (7.2)	12 (7)	0.668
Chest tube insertion	4	10	7	
Bleeding	4 (3.7)	12 (6.2)	1 (0.6)	0.002
Severe	0	0	0	
Moderate	3	1	1	
Minor	1	11	0	
Respiratory failure	0	0	0	
Death	0	0	0	

Abbreviations: ENB, electromagnetic navigation bronchoscopy; RP‐EBUS, radial probe endobronchial ultrasound.

## DISCUSSION

In this study, the combination of ENB and RP‐EBUS significantly increased the diagnostic yield of lesions with a solid part less than 20 mm and the presence of type II or III bronchus signs. This would be a useful reference when selecting bronchoscopies, especially in low‐ and middle‐income countries where it is difficult to readily access the all of the equipment.

This study had a diagnostic yield of 63.5%, which is very meaningful considering that all procedures were performed under moderate conscious sedation, and that only nodules evaluated as unfeasible for biopsy by PCNB were included. This is consistent with or slightly higher than ENB diagnostic yields of 49–63.3% using the strict definition reported by previous large prospective studies.^20^, ^21^ A recent prospective trial that used the same ENB model as that used in this study (SPiN Thoracic Navigation System; Veran Medical) reported a diagnostic yield of 49.3% for ENB.^22^

Recently, remarkable advancements in diagnostic bronchoscopy have prompted many studies on the diagnostic value of advanced bronchoscopy. However, the lack of a standardized method for the diagnostic yield calculation has made it challenging to compare across studies and assess the diagnostic value of bronchoscopy. The diagnostic yield differs even within the same study, depending on the methods applied.^18^, ^23^ In the era of rapid advancements in bronchoscopies, rather than waiting for a follow‐up period of more than 1 year, calculating the diagnostic yield using the results obtained at the index procedure has been suggested. In clinical practice, physicians make decisions based on the immediate result of the procedure.^19^ In this context, Vachani et al. recently proposed three methods for computing the diagnostic yield, and our study aligns with these efforts by clarifying the diagnosis of all cases and clearly defining the diagnostic yields using the three methods.^18^

Although the diagnostic yields following the combination of ENB and RP‐EBUS were not different, the lack of specific criteria for allocating patients and the sole determination of a combination based on the operator's decision may have led to a preference for the combination in technically challenging cases, therefore the potential value of the combination may have been underestimated. Moreover, the observation of smaller nodule sizes in the combination group suggests a possible inclination toward using the combination in challenging cases. Given that most of the previous literature has already shown that combining the two methods leads to better results but also increases costs and complication rates, we hypothesized that the combination may be unnecessary for simple cases.^13^, ^14^, ^15^, ^16^ Hence, it was identified that the characteristics correlated with a reduced diagnostic yield, and an additional subgroup analysis was conducted by specifically selecting nodules based on the outcomes of the multivariable analysis. Consequently, it was observed that the combination of the two methods increased the diagnostic yield for nodules with a solid component <20 mm and the presence of type II or III bronchus signs. However, other than that, there was no advantage from the combination, and rather the complication rates increased, indicating that careful selection is required.

This study showed that the risk of bleeding increased in the combination group. Furthermore, the risk of bleeding decreased in the RP‐EBUS group compared with the ENB group, probably because the guide sheath (GS) used during RP‐EBUS aids hemostasis by blocking the bronchus. In the combination group, the lesion was only evaluated using ultrasound, not GS, and a biopsy was performed using ENB. Consequently, the complication rates of the combination group were expected to be similar to those of the ENB group, but the higher bleeding rate in the combination group may be attributed to the additional stimulation incurred during the exploration of the lesion via an ultrasound probe. Moreover, obtaining tissue samples in a more definitive solid area validated by the radial probe may contribute to a relatively higher tendency for bleeding. However, further well‐controlled prospective studies are needed to confirm these findings.

There are several limitations to this study. First, as this was a single‐center, retrospective study, there may have been selection bias, therefore, to confirm the findings of this study, a large prospective study is required. Second, the follow‐up period was relatively short, at 6 months. If the follow‐up period was more than a year, the diagnostic yields in the intermediate and liberal definitions could have increased. Third, the decision to combine solely depended on the operators, and the results showed that they had a tendency to combine both methods for nodules with less solid parts. In these cases, combination therapy increased diagnostic yields more than the ENB or RP‐EBUS alone groups. This paradoxically demonstrates that the operators' clinical judgment was effective according to our data. Alternatively, the results from the actual data support the operators' clinical hypothesis regarding the circumstances in which combination therapy would be beneficial.

This study demonstrated that the combination of ENB and RP‐EBUS provides a higher diagnostic yield for nodules with type II or III bronchus signs and a smaller solid part, therefore a suitable selection for the combination should be made on the basis of the features of each nodule, especially where access to various equipment is limited.

## AUTHOR CONTRIBUTIONS

Conceptualization: Lee B, Choi C‐M, Ji W. Data curation: Lee B, Ji W. Formal analysis: B Lee, Hwang HS, Jang SJ, Oh SY, Kim MY, Choi C‐M, Ji W. Investigation: Lee B, Ji W. Methodology: Lee B, Ji W. Visualization: Lee B. Supervision: Hwang HS, Jang SJ, Oh SY, Kim MY, Choi C‐M, Ji W. Writing–original draft: Lee B, Ji W. Writing–review & editing: Hwang HS, Jang SJ, Oh SY, Kim MY, Choi C‐M, Ji W.

## CONFLICT OF INTEREST STATEMENT

The authors have no competing interests to declare.

## Supporting information


**SUPPORTING INFORMATION TABLE S1** The contingency table of diagnostic results from total bronchoscopy procedures
**SUPPORTING INFORMATION TABLE S2** Multivariable analysis for the diagnostic yield
**SUPPORTING INFORMATION TABLE S3A** The multivariable analysis for the diagnostic yield in solid nodules
**SUPPORTING INFORMATION TABLE S3B** The multivariable analysis for the diagnostic yield in non‐solid nodules
