# Supplementary material for: Optimal approach for diagnosing peripheral lung nodules by combining electromagnetic navigation bronchoscopy and radial probe endobronchial ultrasound
Source: Thorac Cancer. 2024 Jun 17;15(21):1638–45. doi: 10.1111/1759-7714.15376 (PMC11260552; doi:10.1111/1759-7714.15376)
Supplement: Supplementary file 1 — SUPPORTING INFORMATION TABLE S1 The contingency table of diagnostic results from total bronchoscopy procedures SUPPORTING INFORMATION TABLE S2 Multivariable analysis for the diagnostic yield SUPPORTING INFORMATION TABLE S3A The multivariable analysis for the diagnostic yield in solid nodules SUPPORTING INFORMATION TABLE S3B The multivariable analysis for the diagnostic yield in non‐solid nodules [file TCA-15-1638-s001.docx]

**Supplementary Table 1.** The contingency table of diagnostic results from total bronchoscopy procedures

|  | NSCLC | SCLC | Other primary lung cancer or unknown type | Metastatic cancer | lymphoma | Granuloma | OP | INF-TB/NTM | INF-fungus | INF-bacteria or pathogen not identified | Benign lung tumor* | ILD | Therapeutic procedure without confirmation of diagnosis or lesion increased | lesion regressed | stationary | f/u loss | Total |
| --- | --- | --- | --- | --- | --- | --- | --- | --- | --- | --- | --- | --- | --- | --- | --- | --- | --- |
| NSCLC | 231 |  |  |  |  |  |  |  |  |  |  |  |  |  |  |  | 231 |
| SCLC |  | 3 |  |  |  |  |  |  |  |  |  |  |  |  |  |  | 3 |
| Other primary lung cancer or unknown type |  |  | 9 |  |  |  |  |  |  |  |  |  |  |  |  |  | 9 |
| Metastatic cancer in the lung |  |  |  | 8 |  |  |  |  |  |  |  |  |  |  |  |  | 8 |
| lymphoma |  |  |  |  | 5 |  |  |  |  |  |  |  |  |  |  |  | 5 |
| Granuloma without organisms |  |  |  |  |  | 4 |  |  |  |  |  |  | 1 |  |  |  | 5 |
| Organizing pneumonia (OP) | 2 |  |  |  |  |  | 16 |  |  |  |  |  | 1 |  |  | 1 | 20 |
| Infection-TB/NTM |  |  |  |  |  |  |  | 10 |  |  |  |  |  |  |  |  | 10 |
| Infection-fungus |  |  |  |  |  |  |  |  | 10 |  |  |  |  |  |  |  | 10 |
| Infection-bacteria or pathogen not identified |  |  |  |  |  |  |  |  |  | 15 |  |  |  |  |  |  | 15 |
| Benign lung tumor* |  |  |  |  |  |  |  |  |  |  | 1 |  |  |  |  |  | 1 |
| ILD** |  |  |  |  |  |  |  |  |  |  |  | 12 | 1 |  |  | 1 | 14 |
| Non-neoplastic lung parenchyma / bronchial tissue | 66 | 2 | 3 | 2 | 1 |  |  |  | 3 | 1 | 1 | 4 | 17 | 8 | 20 | 23 | 151 |
| A few atypical cells*** | 6 |  | 1 |  | 1 |  |  |  |  |  |  |  | 4 |  | 1 | 2 | 15 |
| Nonspecific inflammation**** | 13 |  |  |  |  | 1 | 2 |  |  | 1 | 1 | 1 | 2 | 2 | 3 | 5 | 31 |
| Focal alveolar epithelial proliferation |  |  |  |  |  |  |  |  |  |  |  |  |  |  | 1 |  | 1 |
| Total | 318 | 5 | 13 | 10 | 7 | 5 | 18 | 10 | 13 | 17 | 3 | 17 | 26 | 10 | 25 | 32 | 529 |

†Sclerosing pneumocytoma, granular cell tumor, capillary hemangioma

‡Fibrotic sequelae, sarcoidosis, IgG4-related disease, amyloidosis, progressive massive fibrosis

§Unknown significance or atypical pneumocytic hyperplasia, atypical adenomatous hyperplasia

¶Acute inflammation without granulomas, Chronic inflammation, lymphocytic interstitial infiltration, anthracofibrosis

**Supplementary Table 2.** Multivariable analysis for the diagnostic yield

|  | Adjusted OR (95% CI) | *P* value |
| --- | --- | --- |
| **Combination** | 1.19 (0.81 – 1.74) | 0.37 |
| **Solidity** |  |  |
| Solid | Ref |  |
| Part-solid or ground-glass opacity | 1.23 (0.81 – 1.88) | 0.33 |
| **Size of solid part** |  |  |
| ≤ 20 mm | Ref |  |
| > 20 mm | 1.62 (1.08 – 2.43) | 0.02 |
| **Location (central vs. peripheral)** |  |  |
| I (inner third) | Ref |  |
| II (middle third) | 0.68 (0.41 – 1.12) | 0.12 |
| III (outer third) | 0.97 (0.61 – 1.55) | 0.89 |
| **Bronchus sign** |  |  |
| Type I | Ref |  |
| Type II or III | 0.68 (0.44 – 1.04) | 0.07 |

OR = odds ratio, Ref = reference value

**Supplementary Table 3A.** The multivariable analysis for the diagnostic yield in solid nodules

|  | Adjusted OR (95% CI) | *P* value |
| --- | --- | --- |
| **Combination** | 0.77 (0.48 − 1.23) | 0.28 |
| **Size of solid part** |  |  |
| ≤ 20 mm | Ref |  |
| > 20 mm | 1.11 (0.69 − 1.80) | 0.66 |
| **Location (central vs. peripheral)** |  |  |
| I (inner third) | Ref |  |
| II (middle third) | 0.58 (0.31 − 1.07) | 0.08 |
| III (outer third) | 0.91 (0.52 − 1.58) | 0.73 |
| **Bronchus sign** |  |  |
| Type I | Ref |  |
| Type II or III | 0.67 (0.41 − 1.07) | 0.09 |

**Supplementary Table 3B. The multivariable analysis for the diagnostic yield in non-solid nodules**

|  | Adjusted OR (95% CI) | *P* value |
| --- | --- | --- |
| **Combination** | 2.56 (1.34 − 4.89) | 0.004 |
| **Size of solid part** |  |  |
| ≤ 20 mm | Ref |  |
| > 20 mm | 5.09 (2.18 − 11.89) | < 0.001 |
| **Location (central vs. peripheral)** |  |  |
| I (inner third) | Ref |  |
| II (middle third) | 1.32 (0.54 − 3.22) | 0.54 |
| III (outer third) | 1.71 (0.71 − 4.10) | 0.23 |
| **Bronchus sign** |  |  |
| Type I | Ref |  |
| Type II or III | 0.67 (0.41 − 1.07) | 0.80 |
